# Sex differences in abnormal gluten response and predictors of gluten sensitivity in patients with schizophrenia

**DOI:** 10.3389/fimmu.2025.1696763

**Published:** 2025-11-04

**Authors:** Michał Dzikowski, Joanna Rog, Dariusz Juchnowicz, Anna Rymuszka, Hanna Karakula-Juchnowicz

**Affiliations:** ^1^ I Department of Psychiatry, Psychotherapy and Early Intervention in Lublin, Medical University of Lublin, Lublin, Poland; ^2^ Department of Basic Medical Sciences, Faculty of Medicine, The John Paul II Catholic University of Lublin, Lublin, Poland; ^3^ Department of Psychiatry and Psychiatric Nursing, Medical University of Lublin, Lublin, Poland; ^4^ Department of Animal Physiology and Toxicology, Faculty of Medicine, The John Paul II Catholic University of Lublin, Lublin, Poland

**Keywords:** inflammation-dependent psychiatric disorders, gluten, cytokines, low-grade inflammation, food immune reactivity

## Abstract

**Introduction:**

Immune-inflammatory dysregulations are linked to shifts in specific gut microbiota genera, underscoring the importance of the gut–brain connection in schizophrenia (SZ). However, the immune-inflammatory aspects of sex differences in SZ remain largely unexplored. The aims of this study were (1) to identify sex-related differences in inflammatory response, intestinal biomarkers, and gluten sensitivity in SZ and (2) to determine potential factors underlying variability in the immune response to gluten in this population.

**Methods:**

A total of 102 individuals with SZ and 60 healthy controls (HC) were included in the study.

**Results:**

Elevated titers of anti-gliadin (AGA) IgA were found in 26% of individuals with SZ compared to 22% of HC and elevated AGA IgG in 30% of SZ patients compared to 20% of HC. The IgG levels were higher in men than in women, regardless of health status. Significant differences in the levels of AGA IgG and deamidated gliadin (dGP) IgG were observed between men and women with SZ, with higher concentrations detected in men. Factors differentiating patients with positive AGA IgA antibodies included tissue transglutaminase (tTG) IgA levels, high-sensitivity C-reactive protein (hs-CRP) levels, and age. Factors associated with positive AGA IgG antibodies included dGP and anti-*Saccharomyces cerevisiae* (ASCA) antibody levels, negative symptoms of SZ, and age of onset.

**Discussion:**

This is the first study to examine sex-related differences and illness stage in the immune response to gluten among SZ patients. Stronger inflammatory responses were found in men, suggesting sex-related disparities in gluten-related immune activation. These findings highlight a complex interplay between hormones, immune function, intestinal barrier integrity, and psychiatric symptoms. Further longitudinal research is needed to clarify these mechanisms and their clinical significance.

## 1 Introduction

Schizophrenia (SZ) is a chronic, multidimensional disorder characterized by psychotic, negative, cognitive, mood, and motor symptoms of varying severity. A paper published in 2024 summarized the current findings on SZ and identified immune aberrations and inflammation as etiopathological factors of the disorder (1). A meta-analysis of 69 studies indicates the presence of abnormal inflammatory markers in the cerebrospinal fluid (CSF) of patients with schizophrenia (SZ) compared to healthy individuals, suggesting increased blood–brain barrier (BBB) permeability. The immune system is considered a potential mediator in the development of psychosis (2). Immune-inflammatory dysregulation, not limited to the brain, also affects other organs and systems, potentially influencing disease progression and the physical condition of the patient. Altered levels of immune-inflammatory markers have been associated with differentially abundant genera in the fecal microbiome, highlighting the importance of the gut–brain connection in the course of schizophrenia (3). Several studies have identified elevated levels of pro-inflammatory cytokines in SZ, and reducing inflammation has been proposed as a potential therapeutic strategy to alleviate symptoms and improve overall clinical outcomes. Additionally, immune responses to dietary antigens have been implicated in modulating gut microbiota composition and systemic inflammation. These findings suggest that interactions between peripheral immune activation and microbial imbalance may contribute to both psychiatric symptomatology and physical comorbidities in SZ (4).

Psychopathological symptoms and cognitive impairment in patients with SZ may be the result of certain bacteria, mediated by subclinical inflammation and impaired intestinal permeability (5, 6). Changes in intestinal permeability have been proposed as a potential cause of inflammation in SZ. The mucosal barrier of the gastrointestinal tract plays a critical role in digestion, absorption, and metabolism and mediates interactions between the host and the external environment. Exposure to harmful factors can increase gut permeability and activate immune-inflammatory pathways (7). Gliadin is a component of wheat gluten, a well-known dietary factor that intensifies intestinal paracellular permeability. This protein exacerbated the effect of other factors engaged in intestinal mucosa damage (8, 9). Approximately 30% of patients with SZ are characterized by elevated titers of antibodies against gluten. The immune response to gluten in SZ is both shared with and distinct from the response observed in celiac disease (10). Anti-gliadin antibodies (AGA) IgG are correlated with both peripheral and central markers of inflammation in patients with SZ (11, 12). The mechanistic basis linking SZ, the gut–brain axis, and inflammation remains poorly understood. According to available data, differences in inflammatory-immune pathways were associated with higher levels of inflammation and poorer immune functioning only in the female subgroup (13–15). Despite the connection between immunological pathways and schizophrenia symptoms, men show worse outcomes (1). The better prognosis observed in women may be related to the psychoprotective role of estrogens (16). Scientific understanding of sex-related differences in SZ remains limited and inconsistent.

The immune-inflammatory aspects of sex differences in SZ are rarely studied. The aim of the study was to analyze sex-related differences in the inflammatory response, intestinal biomarkers, and gluten sensitivity in patients with SZ as well as the potential factors contributing to this variability.

## 2 Materials and methods

The study complies with the provisions of the Declaration of Helsinki (17), was conducted in accordance with all relevant local legislation, and was approved by the Ethics Committee of the Medical University of Lublin, Poland (project identification code: KE-0254/231/2013). Prior to enrollment, all potential participants were informed about the aim, methodology, voluntary nature of participation, and the possibility of withdrawing from the study at any time. Informed consent was obtained through a signed written consent form.

A total of 102 patients who met the criteria for schizophrenia (SZ) according to *the Diagnostic and Statistical Manual of Mental Disorders, Fifth Edition* (DSM-5) were recruited as the SZ group. The control group (HC) consisted of 60 healthy individuals.

The inclusion criteria for the SZ group were as follows:

1. signed informed consent for participation in the study
2. diagnosis of SZ according to DSM-5

The exclusion criteria for the SZ group were as follows:

1. lack of signed informed consent
2. neurological disorders, intellectual disability, organic brain dysfunction, or addiction (except for nicotine and caffeine)
3. celiac disease or other autoimmune disorders
4. inflammatory diseases or any other condition in an unstable phase
5. clinical signs of inflammation (high-sensitivity C-reactive protein [hs-CRP] >5 µg/mL) and/or leukocytosis (>10,000/µL) at the time of study admission
6. pregnancy or lactation (in women)

The exclusion criteria for the control group were similar to those of the SZ group, except for the presence of current or past psychiatric disorders.

### 2.1 Sociodemographic and clinical data

The symptomatology of the SZ group was assessed by a well-trained psychiatrist with experience in diagnosing and treating psychotic disorders. The following information was collected from the patients: age, sex, age at onset, duration of illness, number of hospitalizations, current and past comorbidities, pharmacological treatment, body weight, and height. The same information—excluding data related to the disease—was also collected from the HC group.

In the SZ group, symptom severity was assessed using the *Positive and Negative Syndrome Scale* (PANSS) in Polish adaptation (18) at the day of blood assessment. Antipsychotic medication doses were standardized using defined daily doses (DDDs) and expressed in olanzapine equivalents (1 mg) according to Leucht et al. (19). Based on obtained clinical information, the SZ group was divided into a first-episode schizophrenia (FEP) group and a chronic schizophrenia (CS) group, with the cutoff point set at 24 months after the first treatment contact.

### 2.2 Gluten intake

The assessment of gluten consumption was carried out using the food frequency method, as described in our previous study (20). The questionnaire used in the analysis was based on the validated *Food Frequency Questionnaire with six answers* (FFQ-6) (21). Portion sizes of consumed foods were estimated using the *Album of Photographs of Food Products and Dishes* (22). The amount of gluten from each cereal source was determined by multiplying the protein content of gluten-containing cereals by 0.8, and the results were summed to express the total gluten intake in grams per day per person (23, 24).

### 2.3 Blood collection and laboratory analysis

Venous blood samples were drawn in the morning following an overnight fast. To obtain serum, the samples were centrifuged (at 2,000 ×*g* for 10 min at room temperature) and stored at −80 °C until the day of the enzyme-linked immunosorbent assay (ELISA) analysis.

The serum levels of gliadin IgA and IgG antibodies (AGA), transglutaminase IgA antibodies (tTG), deamidated gliadin peptide IgG antibodies (dGP), interleukin-6 (IL-6), high-sensitivity C-reactive protein (hs-CRP), anti-*Saccharomyces cerevisiae* antibodies (ASCA), and soluble cluster of differentiation 14 (sCD14) were assessed using the ELISA method with commercially available kits (see **Table 1**).

**Table 1:** Summary of the examined biomarkers: source and clinical relevance.

Examined factor	Source	Role/importance
AGA IgA antibodies	Demeditec Diagnostics GmbH	Non-specific antibodies associated with celiac disease can also be detected in other conditions, typically disappearing after 3–9 months on a gluten-free diet (51, 52)
AGA IgG antibodies	Demeditec Diagnostics GmbH	The most commonly detected antibodies in non-celiac gluten sensitivity, typically disappearing after 3–9 months on a gluten-free diet (51, 52)
tTG antibodies IgA	BluWell	Potential basic tool for celiac disease serological testing (53)
dGP antibodies IgG	BlueWell	Potential basic tool for celiac disease serological testing (53)
IL-6	BlueWell	Cytokine-mediated inflammation in non-communicable diseases (54)
hs-CRP	Becton, Dickinson and Company BD Biosciences	A positive phase indicator of the ongoing inflammatory in non-communicable diseases (55)
ASCA	Demeditec Diagnostics GmbH	Marker of inflammatory bowel diseases, intestinal inflammation, and candidate biomarker of autoimmune disease (56)
sCD14	R&D Systems	Proposed marker of bacterial infections, immune-inflammation, and gastrointestinal permeability (57)

AGA, anti-gladin antibodies; tTG, anti-tissue transglutaminase antibodies; dGP, anti-deamidated gliadin antibodies; IL-6, interleukin 6; hs-CRP, high sensitivity C-reactive protein; ASCA, anti-*Saccharomyces cerevisiae* antibodies; sCD14, soluble cluster of differentiation 14.

### 2.4 Statistical analysis

Shapiro–Wilk test was used to assess the normality of data distribution before group comparisons. Since the data were not normally distributed, non-parametric tests were applied. Mann–Whitney *U*-test was used to detect differences between women and men, and Spearman rank correlation test was performed to assess correlations and their directions. Categorical variables were presented as frequencies and percentages, while ordinal and continuous variables were presented as medians with minimum and maximum values. Chi-square test was used to evaluate differences between categorical variables.

Potential factors affecting gluten sensitivity in female and male patients were identified using the Classification and Regression Tree (CART) method. This approach allows for the classification of patients based on the identified factors, enabling a more tailored understanding of gluten sensitivity patterns across sexes. Statistical analysis further supports the differentiation of risk profiles and potential predictors in each subgroup. Statistical analyses were performed using Statistica 13 software (StatSoft, Inc., Tulsa, OK, USA). A *p*-value of <0.05 was considered statistically significant.

## 3 Results

### 3.1 Baseline characteristics of the examined group

We did not find any significant differences in age or BMI between the HC and SZ groups (*p* > 0.05). The SZ group had a higher gluten intake compared to the HC group (*p* = 0.01). Women from the HC group consumed less gluten (*p* < 0.001) and had lower BMI (*p* < 0.001) compared to men from the same group. Women with SZ were older than men with SZ (*p* = 0.025). However, after subgroup analysis, this age difference remained significant only in the FEP subgroup, where women were older than men (*p* = 0.037). No significant differences in the severity of psychopathological symptoms were found between patient subgroups (*p* > 0.05).

### 3.2 Sex-related differences in gluten sensitivity, intestinal permeability, and inflammatory biomarkers

Significant differences in the levels of AGA IgG (*p* = 0.014) and dGP IgG (p = 0.009) were observed between women and men with SZ, with higher concentrations found in men. In the CS subgroup, women had lower levels of both AGA IgG (*p* = 0.046) and dGP IgG (*p* = 0.011) compared to men. No significant sex-related differences in the levels of the assessed biomarkers were found in the HC and FEP groups (*p* > 0.05). The differences in biomarker levels between men and women are presented in **Table 2**.

**Table 2:** Differences in the examined biomarkers in healthy individuals and patients with schizophrenia.

Biomarker	Me (min–max)		*P*
	F	M	
SZ			
AGA IgA U/mL	6.64 (2.82–22.81)	6.26 (2.24–22.40)	0.457
AGA IgG U/mL	**3.59 (1.51–47.46)**	**5.62 (1.52–220.87)**	0.014*
tTG IgA U/mL	16.25 (3.97–38.56)	15.46 (0.00–45.59)	0.292
dGP IgG U/mL	**5.36 (0.00–41.63)**	**11.41 (0.24–279.88)**	0.009*
ASCA U/mL	0.00 (0.00–18.59)	2.44 (0.00–33.78)	0.102
sCD14 pg/mL	1,746.42 (981.38–5,100.00)	1,607.58 (724.12–3,040.00)	0.114
hs-CRP µg/mL	1.08 (0.00–13.89)	0.60 (0.00–14.60)	0.757
IL-6 pg/mL	4.89 (0.00–32.78)	3.87 (0.00–34.33)	0.271
HC			
AGA IgA U/mL	5.64 (1.42–13.24)	6.87 (2.40–39.43)	0.194
AGA IgG U/mL	4.52 (1.71–63.66)	5.07 (1.57–341.33)	0.743
tTG IgA U/mL	15.65 (4.95–39.59)	16.89 (5.79–45.62)	0.743
dGP IgG U/mL	5.21 (0.00–100.02)	8.19 (0.75–131.28)	0.085
ASCA U/mL	3.54 (0.00–19.85)	4.71 (0.00–137.36)	0.340
sCD14 pg/mL	1,563.88 (260.34–2,460.00)	1,563.88 (708.54–2,700.00)	0.988
hs-CRP µg/mL	0.34 (0.00–4.18)	0.60 (0.00–12.14)	0.109
IL-6 pg/mL	3.02 (0.49–45.27)	3.10 (0.41–13.53)	1.000
FEP			
AGA IgA U/mL	6.04 (2.82–22.53)	6.18 (3.17–22.40)	0.382
AGA IgG U/mL	4.20 (1.80–47.46)	6.01 (1.74–220.87)	0.197
tTG IgA U/mL	15.21 (3.97–34.14)	15.55 (5.35–32.75)	0.993
dGP IgG U/mL	8.04 (1.01–41.63)	11.26 (0.24–279.88)	0.232
ASCA U/mL	0.00 (0.00–17.78)	1.47 (0.00–16.74)	0.160
sCD14 pg/mL	1,707.32 (981.38–2,200.00)	1,524.65 (837.66–3,040.00)	0.119
hs-CRP µg/mL	0.59 (0.00–7.36)	0.43 (0.00–14.60)	0.978
IL-6 pg/mL	4.04 (0.71–21.57)	3.08 (0.00–16.96)	0.128
CS			
AGA IgA U/mL	7.35 (3.00–22.81)	6.38 (2.24–16.66)	0.185
AGA IgG U/mL	**3.00 (1.51–11.96)**	**4.57 (1.52–101.98)**	0.046*
tTG IgA U/mL	21.04 (7.20–38.56)	14.52 (0.00–45.59)	0.154
dGP IgG U/mL	**4.22 (0.00–27.58)**	**13.39 (0.49–93.75)**	0.011*
ASCA U/mL	1.36 (0.00–18.59)	4.13 (0.00–33.78)	0.306
sCD14 pg/mL	1,905.32 (1,304.06–5,100.00)	1,766.38 (724.12–2,560.00)	0.399
hs-CRP µg/mL	1.50 (0.04–13.89)	1.12 (0.09–11.32)	0.714
IL-6 pg/mL	5.35 (0.00–32.78)	4.55 (0.74–34.33)	0.866

AGA, anti-gladin antibodies; tTG, anti-tissue transglutaminase antibodies; dGP, anti-deamidated gliadin antibodies; IL-6, interleukin 6; hs-CRP, high sensitivity C-reactive protein; ASCA, anti-*Saccharomyces cerevisiae* antibodies; sCD14, soluble cluster of differentiation 14; U, unit; µg, microgram; pg, picogram; mL, milliliter; Me, median; min, minimum; max, maximum; HC, healthy control; SZ, schizophrenia; FEP, first episode of schizophrenia; CS, chronic schizophrenia; F, female; M, males (according to Mann–Whitney *U*). Bold font indicates statistically significant results.

### 3.3 Relationship between sociodemographic data and examined biomarkers depending on sex

In the HC group, age was positively correlated with IL-6 levels in men (*R* = 0.44, *p* < 0.05), negatively correlated with ASCA levels in women (*R* = –0.37, *p* < 0.05), and positively correlated with hs-CRP levels in women (*R* = 0.44, *p* < 0.05). In both male and female subgroups of the HC group, hs-CRP levels were positively associated with BMI (*R* = 0.51, *p* < 0.05). Additionally, in women, dGP levels were positively correlated with BMI (*R* = 0.38, *p* < 0.05).

The connection between examined markers and age was found to have weak significance in men from the SZ group regarding sCD14 (*R* = 0.28, *p* < 0.05) and hs-CRP (*R* = 0.33, *p* < 0.05). After subgroup analysis, a relationship was found in the CS men group between age and ASCA (*R* = 0.52, *p* < 0.05) only. In men with SZ, we found an inverse relationship between smoking and IL-6 concentration (*R* = -0.28; *p* < 0.05), and the relationship was found only in the FEP subgroup (*R* = -0.39, *p* < 0.05).

In women with SZ, age was positively correlated with AGA IgA (*R* = 0.45, *p* < 0.05), AGA IgG (*R* = 0.37, *p* < 0.05), sCD14 (*R* = 0.32, *p* < 0.05), hs-CRP (*R* = 0.47, *p* < 0.05), and tTG IgA (*R* = 0.37, *p* < 0.05). After subgroup analysis, these correlations remained significant only in CS women for AGA IgA (*R* = 0.59, *p* < 0.05), AGA IgG (*R* = 0.47, *p* < 0.05), sCD14 (*R* = 0.46, *p* < 0.05), and hs-CRP (*R* = 0.47, *p* < 0.05). Additionally, in CS women, age was also positively correlated with IL-6 (*R* = 0.44, *p* < 0.05).

The relationship between hs-CRP and BMI was found to be significant only in CS women (*R* = 0.49, *p* < 0.05). In CS women, gluten intake was positively associated with sCD14 (*R* = 0.41, *p* < 0.05), and smoking was positively associated with tTG IgA (*R* = 0.45, *p* < 0.05).

### 3.4 Prevalence of gluten sensitivity in women and men

In our previous paper, we addressed the prevalence of gluten sensitivity in patients with schizophrenia, and our findings were consistent with earlier reports (20). Elevated titers of AGA antibodies were found in 26% of individuals with SZ compared to 22% of HC for the AGA IgA subclass and in 30% of individuals with SZ compared to 20% of HC for the AGA IgG subclass. The prevalence of elevated AGA antibody levels in the studied groups is presented in **Table 3**.

**Table 3:** Differences in gluten sensitivity in women and men from the SZ and HC groups.

Group	Gender	Elevated levels (*N*)	Elevated levels (%)
AGA IgA			
SZ, *n* = 102	Female	17	36
	Male	19	36
HC, *n* = 60	Female	6	17
	Male	7	24
AGA IgG			
SZ, *n* = 102	Female	6	13
	Male	24	45
HC, *n* = 60	Female	6	17
	Male	6	24

AGA, antigliadin antibodies; SZ, schizophrenia; HC, healthy control; *N*, number.

Higher levels of AGA IgA and IgG compared to the HC group characterized the SZ group. However, the IgG levels were found to be higher in men than in women regardless of health status.

### 3.5 Potential predictors of gluten sensitivity in patients with schizophrenia

**Figure 1:**
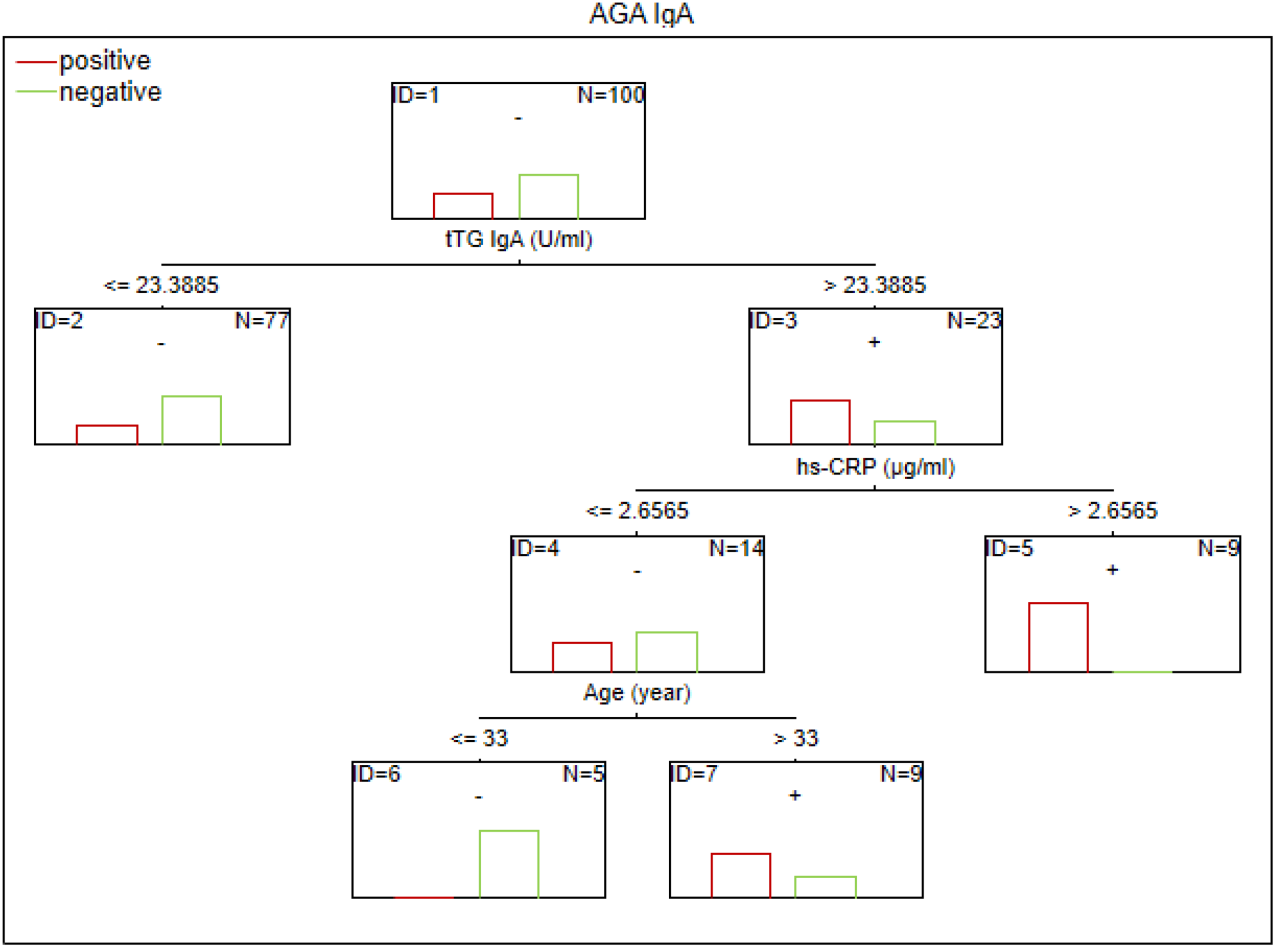
Classification tree generated with the defining characteristics of individuals with SZ and positive AGA IgA antibodies using the C&RT method.

**Figure 2:**
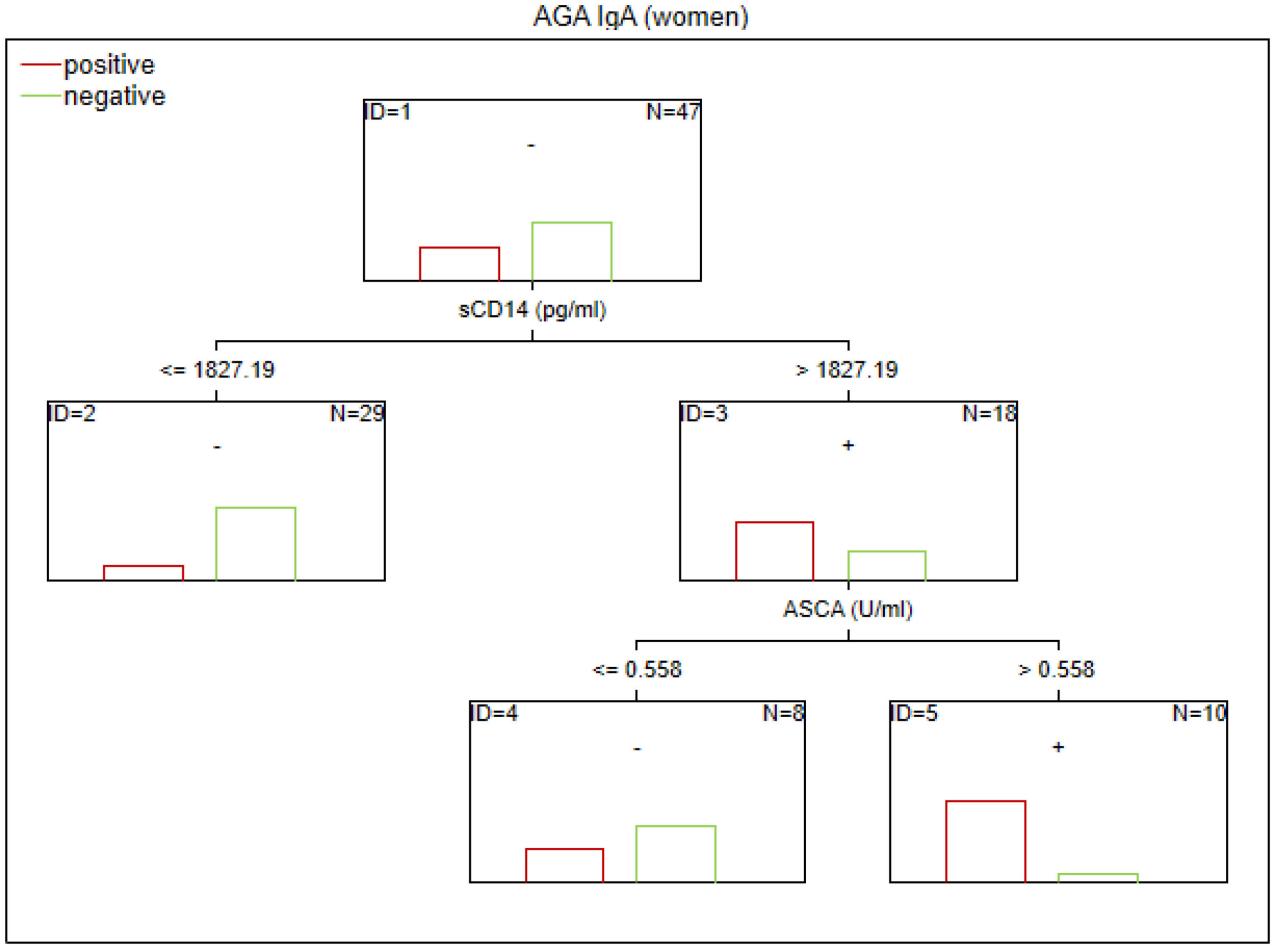
Classification tree generated with the defining characteristics of women with SZ and positive AGA IgA antibodies using the C&RT method.

**Figure 3:**
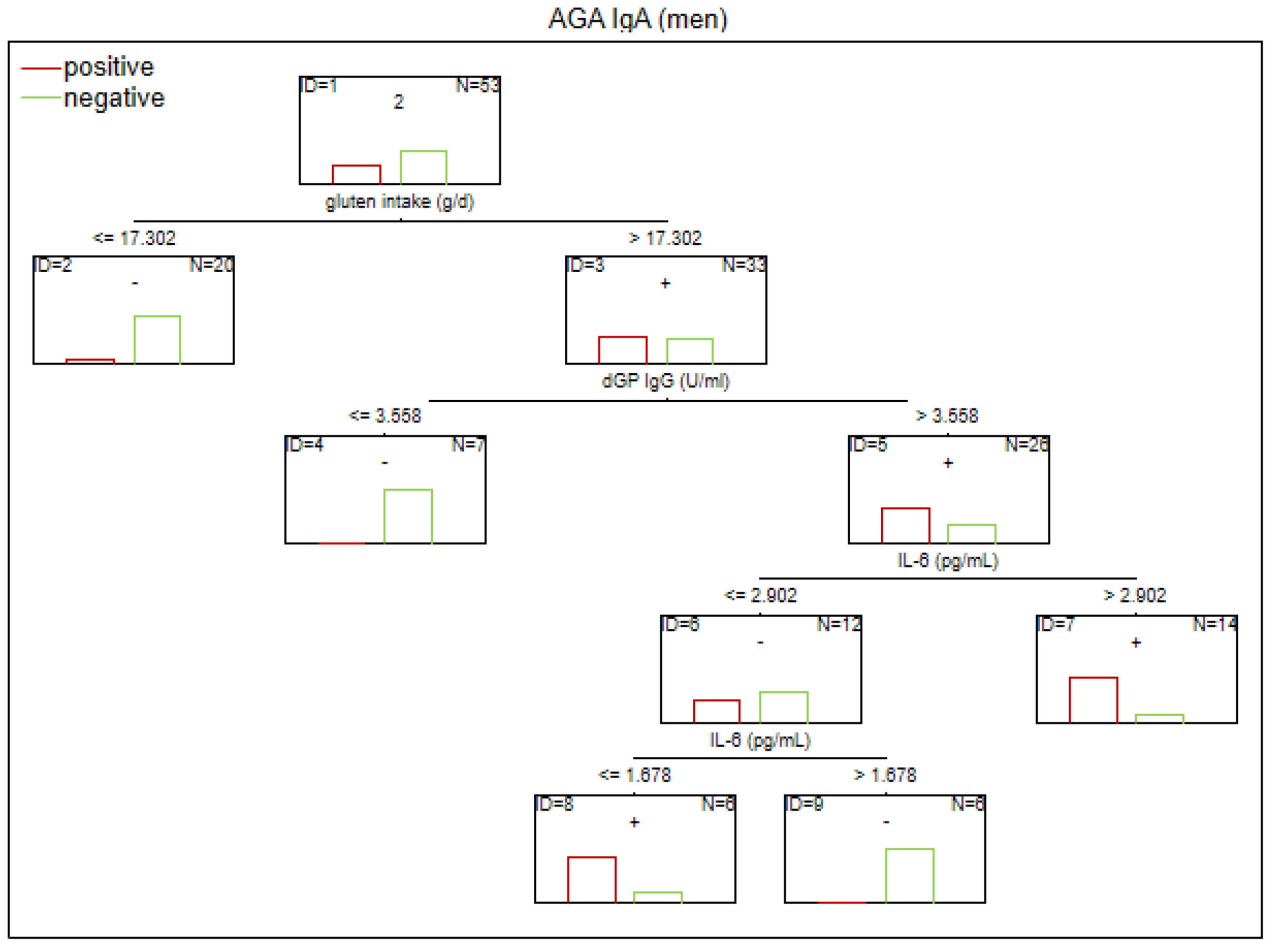
Classification tree generated with the defining characteristics of men with SZ and positive AGA IgA antibodies using the C&RT method.

**Figure 4:**
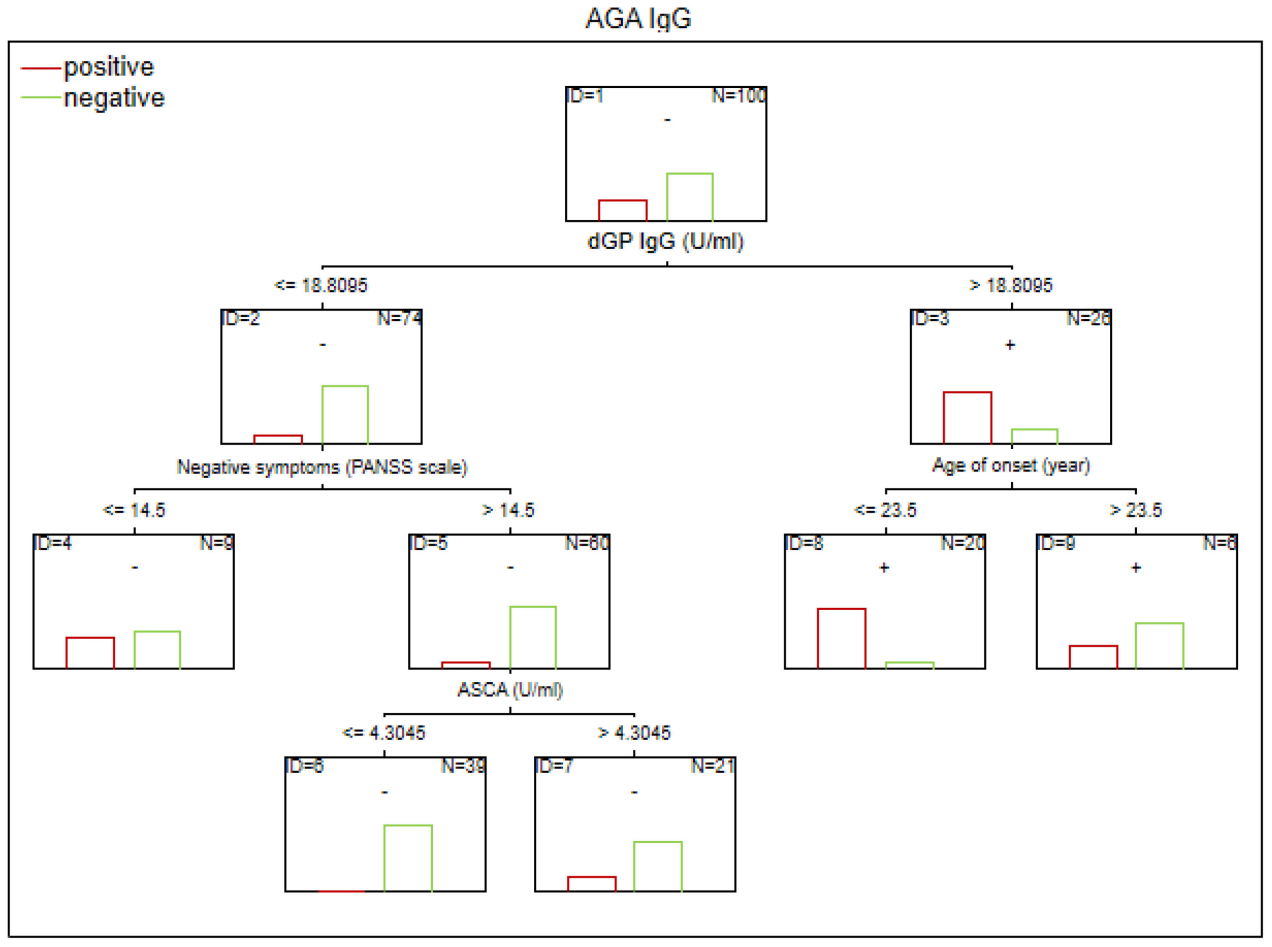
Classification tree generated with the defining characteristics of individuals with SZ and positive AGA IgG antibodies using the C&RT method.

**Figure 5:**
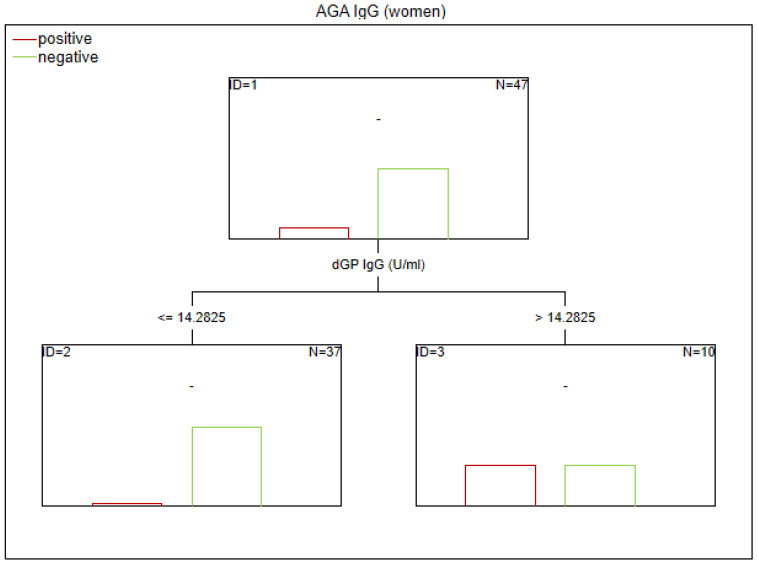
Classification tree generated with the defining characteristics of women with SZ and positive AGA IgG antibodies using the C&RT method.

**Figure 6:**
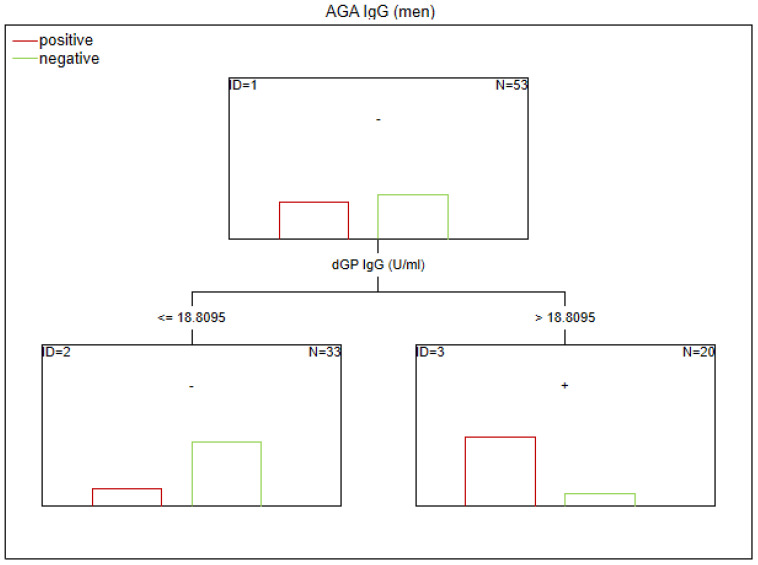
Classification tree generated with the defining characteristics of women with SZ and positive AGA IgG antibodies using the C&RT method.

There are no established biomarkers for the detection of gluten sensitivity. However, elevated levels of AGA IgG and IgA antibodies have been observed, primarily in individuals with abnormal immunological responses to gluten. The CART method was used to determine the potential factors related to the abnormal immune response to gluten in SZ. The considered variables were as follows: age, sex, biological markers, schizophrenia symptoms (positive, negative, general symptoms, and total psychopathology), age of onset, duration of illness, duration of the episode, duration of psychosis, duration of prodrome, number of episodes, DDA, BMI, and gluten intake. Given the observed sex-specific differences in the immune response in SZ, we analyzed sex as a potential factor affecting gluten sensitivity. Separate analyses were also conducted for the male and female subgroups. The results of the CART analysis are depicted in **Figures 1**–**6**.

According to our analysis, the factors that differentiated patients with positive AGA IgA antibodies included tTG IgA levels, hs-CRP levels, and age (in the total group), sCD14 and ASCA levels (in women), and gluten intake, dGP IgG, and IL-6 levels (in men). The area under the receiver operating characteristic curve (AUC ROC) was 0.72 for the total group, 0.79 for women, and 0.47 for men.

Factors associated with positive AGA IgG antibodies were dGP and ASCA levels, negative schizophrenia symptoms, and age of onset (in the total group). In both women and men, the dGP IgG levels were the main differentiating factor, with AUC ROC values of 0.88 for the total group, 0.96 for women, and 0.79 for men. The cutoff points for each variable are presented in **Figures 1**–**6**.

## 4 Discussion

The aims of the study were (1) to identify sex-related differences in inflammatory response, intestinal biomarkers, and gluten sensitivity in patients with schizophrenia and (2) to determine potential factors underlying variability in the immune response to gluten in this population, with particular attention to sex and disease stage.

To date, numerous studies have confirmed an abnormal immune response to gluten in patients with SZ and suggested the potential utility of a gluten-free diet in this subgroup (20, 25–27). However, the factors contributing to heightened immune reactivity to cereal proteins remain poorly understood. According to our analysis, men with SZ exhibited higher levels of AGA IgG compared to women with SZ. Overall, 30% of the schizophrenia group had elevated AGA IgG titers (20). Notably, the prevalence of AGA IgG positivity was 45% in men and 13% in women within the SZ subgroup (in HC: 24% in men and 17% in women). Among women from the CS group, older age was associated with higher AGA IgG levels.

Based on CART analysis, higher levels of dGP IgG were identified as the main predictor of AGA IgG positivity. A younger age of onset was associated with AGA IgG sensitivity among individuals with dGP IgG concentrations ≥18.81 U/mL. Elevated AGA IgG levels in patients with SZ have been reported in numerous studies, and peripheral AGA IgG has been shown to correlate with its CSF concentrations (25, 28, 29). This relationship was observed in patients with SZ but not in healthy individuals, suggesting BBB dysfunction in SZ. Increased BBB permeability may influence the pro/anti-inflammatory state of CNS. In SZ, AGA IgG levels were correlated with a neuroinflammatory profile, as indicated by elevated CNS concentrations of myo-inositol and total choline (30). In SZ patients with positive AGA IgG antibodies, a pro-inflammatory phenotype has been confirmed, which is consistent with our findings (31). This is further supported by evidence of a relationship between peripheral inflammation (TNF-α and IL-1β) and AGA IgG levels. Even antibody levels below the positivity threshold, but still within the higher range, may contribute to the inflammatory response supporting the concept of a continuum of immune activation (32).

Our findings indicate an immuno-gastrointestinal link in the pathophysiology of SZ. ASCA levels, a marker of GI inflammation, >4.30 U/mL predicted AGA IgG positivity and greater severity of negative symptoms in the subgroup of SZ patients who were negative for dGP IgG. Severance et al. found a relationship between ASCA and anti-gluten and casein antibodies in SZ (33). Peripheral IgG antibody levels corresponded to their concentrations in CSF in individuals with SZ (34). The selective diffusion of IgG antibodies may suggest BBB dysfunction in SZ (30).

In contrast to our results, a model study suggests a higher anti-gluten immune response AGA IgG triggered by *T. gondii* infection in women compared to men (35). These findings are consistent with analyses of the Iranian population with celiac disease, where women exhibited higher IgG AGA concentrations (36). However, a recent study involving 26 individuals with SZ-related disorders found elevated AGA IgG levels in 46% of the participants, while 73% of the study population were men. The higher prevalence of an abnormal immune response to gluten, greater than usually observed in the SZ population, may be sex-related (37). The sex hormones (androgens, estrogens, and progestogens) are responsible for mediating differences in immune response between men and women and could partially explain the results of this study. In the further analysis, including levels of sex hormones and information on menstrual cycle in the women subgroup may clarify the issue. Androgens and progestogens have immunomodulatory and immunosuppressive effects, while estrogens enhance humoral immunity response. Estrogens regulate the Th2 immune response, contributing to a more specific and controlled activation (38).

In men, the humoral immune response—particularly involving IgG antibodies against food compounds—may be more intense, less specific, and slower to downregulate. The testosterone importance in the modulation of inflammatory pathways was found. In spite of its broadly immunosuppressive properties, testosterone may contribute to a delayed or insufficient downregulation of the IgG-mediated immune response (39, 40). The replacement therapy of testosterone allows the increase of circulating monocytes, which are responsible for pro-inflammatory and early inflammatory response (41). Gliadin-induced response in monocytes is similar to that stimulated by lipopolysaccharide, suggesting an innate immune component to the response (42, 43). This could lead to a more robust inflammatory and humoral reaction in men, resulting in higher levels of AGA IgG.

It has been shown that men with schizophrenia have an earlier age at onset compared to women (44). A longer duration of immune system engagement and a pro-inflammatory state associated with earlier onset may contribute to increased levels of anti-gluten antibodies. Sex is an important indicator of gut microbiota composition and could help explain differences in antibody levels (45). However, this requires further investigation.

The genetic makeup of women integrates X chromosome contributions from both the mother and the father. Many X-linked genes are involved in immune regulation, including those encoding Toll-like receptors (TLRs), pattern recognition receptors (PRRs), and nuclear factor kappa-light-chain-enhancer of activated B cells (NF-κB) (46). This provides women with potential immunological advantages, particularly in early pathogen recognition and immune signaling. Generally, women exhibit a more robust production of immunoglobulins. However, men have been shown to produce higher levels of IgG4 compared to women following pertussis (whooping cough) vaccination (47).

Immune activation has been linked with more prominent negative symptoms which are predominant in male patients and appears to be cell-type specific among different subsets of T cells (31). Pan-T cells correlated positively, while helper T cells and Tregs correlated negatively with severity of negative symptoms, indicating that different T cell subsets may play opposing roles in the psychopathology (31). In our study, we did not find differences in negative symptoms between the groups (*p* = 0.16). However, the median (Me) was higher in men compared to women (Me = 23.5 *vs*. Me = 21), suggesting that the sample size may have been insufficient to confirm this association.

Despite the considerable depth and multidimensional nature of the aspects we investigated, some limitations of this study should be acknowledged. Firstly, the cross-sectional design made it impossible to determine causality between the immune response to gluten and SZ. The effect of treatment on IgG levels should also be taken into account. It has been shown that, after the first 6 months of clozapine treatment, a reduction in immunoglobulin levels may occur. A relationship between clozapine and secondary antibody deficiency has been suggested (48). It should be noted that the menstrual cycle, which was not considered in our study, may influence gut permeability, immune response, and immunoglobulin concentration (49). Nevertheless, the population examined in our study included individuals taking medications that potentially affect hyperprolactinemia associated with menstrual cycle disruptions, which could introduce significant bias into the findings. Additionally, women taking contraceptives containing estrogens and progestogens may produce fewer pro-inflammatory cytokines (50). In our study, the use of contraceptives was not analyzed, and this factor should be verified in future research.

## 5 Conclusions

To the best of our knowledge, this is the first study to investigate sex differences and illness stage in the immune response to gluten among individuals with SZ.

Our findings point to potential sex-related disparities in gluten-related immune activation, with a notably stronger humoral inflammatory response observed in male patients. These results support the hypothesis of an intricate interplay between hormonal fluctuations, immune system function, intestinal barrier integrity, and psychiatric symptomatology of SZ.

Although research on abnormal immune responses in schizophrenia remains limited, it represents an important and underrecognized aspect of the disease’s pathophysiology. Further longitudinal studies—including hormonal aspects and the interplay between medication, hormones, the immune system, and lifestyle—are warranted to better understand the mechanisms of these interactions and their clinical significance.

## Data Availability

The raw data supporting the conclusions of this article will be made available by the authors, without undue reservation.
